# The association between breast density and breast cancer pathological response to neoadjuvant chemotherapy

**DOI:** 10.1007/s10549-022-06616-1

**Published:** 2022-05-23

**Authors:** C. Cullinane, A. O. Brien, A. Shrestha, E. O. Hanlon, J. Walshe, J. Geraghty, D. Evoy, D. McCartan, E. McDermott, R. Prichard

**Affiliations:** 1 Department of General, Breast and Endocrine Surgery, St Vincent’s University Hospital, Dublin, Ireland; 2 Department of Radiology, St Vincent’s University Hospital, Dublin, Ireland; 3 Department of Medical Oncology, St Vincent’s University Hospital, Dublin, Ireland

**Keywords:** Breast cancer, Breast density, Neoadjuvant chemotherapy, Pathological response

## Abstract

**Purpose:**

Mammographic Density (MD) refers to the amount of fibroglandular breast tissue present in the breast and is an established risk factor for developing breast cancer. The ability to evaluate treatment response dynamically renders neoadjuvant chemotherapy (NACT) the preferred treatment option in many clinical scenarios. Previous studies have suggested that MD can predict patients likely to achieve a pathological complete response (pCR) to NACT. We aimed to determine whether there is a causal relationship between BI-RADS breast composition categories for breast density at diagnosis and the pCR rate and residual cancer burden score (RCB) by performing a retrospective review on consecutive breast cancer patients who received NACT in a tertiary referral centre from 2015 to 2021.

**Methods:**

The Mann–Whitney U Test was used to test for differences between two independent groups (i.e. those who achieved pCR and those who did not). A binary logistic regression model was used to estimate odds ratios (OR) and corresponding 95% confidence intervals (CI) for an association between the independent variables of molecular subtype, MD, histological grade and FNA positivity and the dependant variable of pCR. Statistical analysis was conducted with SPSS (IBM SPSS for Mac, Version 26.0; IBM Corp).

**Results:**

292 patients were included in the current study. There were 124, 155 and 13 patients in the BI-RADS MD category b, c and d, respectively. There were no patients in the BI-RADS MD category a. The patients with less dense breast composition (MD category b) were significantly older than patients with denser breast composition (MD category c, d) (*p* = 0.001) and patients who had a denser breast composition (MD category d) were more likely to have ER+ tumours. There was no significant difference in PgR status, HER2 status, pathological complete response (pCR), FNA positivity, or RCB class dependent upon the three MD categories. A binary logistic regression revealed that patients with HER2-enriched breast cancer and triple-negative breast cancer are more likely to achieve pCR with an OR of 3.630 (95% CI 1.360–9.691, *p* = 0.010) and 2.445 (95% CI 1.131–5.288, *p* = 0.023), respectively.

**Conclusion:**

Whilst dense MD was associated with ER positivity and these women were less likely to achieve a pCR, MD did not appear to independently predict pCR post-NACT.

## Introduction

Breast density refers to the proportion of fibroglandular breast tissue relative to adipose tissue in the breast [1]. Wolfe first described the association between mammographically dense breasts and excess breast cancer risk in 1976 and since then numerous other studies have demonstrated a strong association between mammographic density and the risk of breast cancer [2, 3]. Women with the highest categories of breast density have a 4- to 6-fold increased risk of developing breast cancer in comparison to their counterparts with less dense breasts, although this may also be due to a ‘masking effect’ from surrounding breast tissue [4, 5].

The theory underpinning breast density and breast cancer risk is hypothesised to be due to cancer potentiation through incorporating a larger amount of high-risk epithelial cells that may proceed to malignancy within the suitable microenvironment [6]. The extra-cellular matrix (ECM) and collagen organisation within the breast stroma of dense breasts differ considerably from that of women with less dense breasts. Similarly, the presence of small proteoglycans and stromal matrix regulators such as metalloproteinases is overexpressed in women with mammographically dense breasts which may initiate tumourigenesis [5]. In essence, high mammographic breast density is thought to represent a proliferative and pro-inflammatory breast microenvironment [7].

Neoadjuvant chemotherapy (NACT) for breast cancer was once reserved for locally advanced breast cancer to induce downstaging and render tumours operable, however up to 30% of breast cancer patients may now receive NACT [8, 9]. Although the long-term survival outcomes for patients treated with neoadjuvant chemotherapy are comparable to those treated with adjuvant chemotherapy, the proposed benefits associated with neoadjuvant chemotherapy are numerous [10]. The most stringent definition of a pathological Complete Response (pCR) is complete disappearance of invasive and in situ residual tumour disease in the breast and axilla (ypT0) [11]. The Residual Cancer Burden (RCB) index developed from the M.D. Anderson Cancer Centre (MDACC) is a scoring system used to quantify residual disease following NACT. The RCB index combines pathological findings in the primary tumour bed and the regional lymph nodes to calculate a continuous index or score.

Considering the individualised response to NACT, predictive markers are required to select patients who will derive a clinical benefit from NACT. Previous studies examining the value of mammographic density in predicting pathological response to NACT have yielded conflicting results. The aim of this study was to investigate the association between BI-RADS breast composition categories and pCR/RCB index after NACT in a consecutive cohort of 292 Irish women with breast cancer.

## Methods

### Study population

Ethical approval was granted from the Research Ethics Department at St Vincent’s University Hospital, Reference Number: RCR21-022. All consecutive patients with breast cancer who received neoadjuvant chemotherapy from 2015 to 2020 in an Irish tertiary cancer centre were retrospectively reviewed. Patients who had distant metastatic disease at presentation, neoadjuvant endocrine therapy, bilateral breast cancer, male breast cancer, or prior cancer therapy before NACT were not eligible for inclusion in the study. Only patients treated with chemotherapy and/or human epidermal growth factor receptor 2 (HER2)-targeted therapy were included.

## Study design

Mammographic density was categorised according to the BI-RADS 5th edition at the time of diagnosis and recorded by five radiologists. The BI-RADS score ranged from a to d with category a meaning “the breasts are almost entirely fatty”, category b meaning “scattered fibroglandular density”, category c meaning “heterogeneously dense” and category d meaning “extremely dense”. Six patients did not have their breast density recorded at the time of diagnosis and were categorised retrospectively by a radiologist who was blinded for patient outcomes. Tumour size was calculated using mammography and ultrasound (from the radiology report). In this institution all patients with breast cancer are routinely screened for axillary nodal disease using ultrasonography ± fine needle aspiration (FNA) of indeterminate or suspicious nodes. The Royal College of Pathologists Guidelines are used for classification of fine needle aspirate cytology. C1 is considered inadequate, C2 is considered benign, C3 contains atypical cells, C4 contains cells suspicious for malignancy and C5 contains malignant cells. There were no patients with C3 axilla FNA cytology. Tumour phenotype was recorded from data derived from diagnostic core needle biopsies. Oestrogen Receptor (ER) and Progesterone Receptor (PgR) were considered positive when > 10% of cells showed positive staining. Progesterone receptor staining was not performed routinely in our institute until 2019. HER2 status was assessed by immunohistochemistry (IHC) and Fluorescent in situ Hybridisation (FISH) confirmation in cases with 2+ by IHC. HER2 positivity was defined as either 3+ with IHC and/or amplified with FISH. Patients received NACT according to standard guidelines. Patients with HER2 positivity received HER2 blockade (trastuzumab) concomitantly with NACT. From July 2020 patients received dual-HER2 blockade with pertuzumab and trastuzumab in line with national guidelines. A complete pathological response referred to the absence of any residual invasive or situ disease in the breast and axilla (pCR) following completion of NACT. The residual cancer burden score and class were recorded in the histology report or calculated using the available data. Individual data on menopausal status were not available and therefore arbitrarily the age 52 was used to categorise women into pre- and post-menopausal categories. The average age of menopause in Ireland is 52 and therefore women < 52 were considered pre-menopausal and those aged ≥ 52 were considered post-menopausal [12].

### Statistical analysis

Preliminary analyses indicated that the continuous variables analysed were not normally distributed and thus nonparametric methods of analyses were used. The Mann–Whitney U Test was used to test for differences between two independent groups (i.e. those who achieved pCR and those who did not achieve pCR) on a continuous measure (i.e. age). The Kruskal–Wallis one-way analysis of variance (*h*) was used to test for difference between three or more independent groups (i.e. MD categories *b, c* and *d*) on a continuous measure (i.e. RCB score and age). To explore the relationship between the categorical variables (i.e. pCR, MD Class, RCB class, ER status, PgR status, HER2 status, FNA axillary node status), the Pearson’s chi-squared (*x*^2^) test for independence was used. A binary logistic regression model was used to estimate odds ratios (OR) and corresponding 95% confidence intervals (CI) for an association between the independent variables of molecular subtype (ER positive/HER2 negative, ER positive/HER2 positive, HER2 enriched, Triple Negative), MD, histological grade and FNA positivity and the dependant variable of pCR. Statistical analysis was conducted with SPSS (IBM SPSS for Mac, Version 26.0; IBM Corp Armonk, NY, USA).

## Results

### Patient characteristics

292 patients were included in the current study. There were 124, 155 and 13 patients in the BI-RADS MD category *b*, *c* and *d,* respectively. There were no patients in the BI-RADS MD category *a*. The distribution of patient characteristics according to BI-RADS MD is presented in Table 1. The patients with less dense breast composition (MD category *b*) were significantly older than patients with denser breast composition (MD category *c, d*) (*p* = 0.001). The proportion of patients with ER+ tumours differed significantly amongst the three MD categories (*p* < 0.014), and patients who had a denser breast composition (MD category *d*) were more likely to have ER+ tumours. There was no significant difference in PR status, HER2 status, pathological complete response (pCR), FNA positivity, or molecular subtype amongst the three MD categories (Table 1).

**Table 1:** Patient characteristics according to mammographic density (MD)

MD		*b* (*n* = 124)	*c* (*n* = 155)	*d* (*n* = 13)	*p* value
Age (years)	Median	52	46	46	0.001*
	Mean	53.02	48.28	44.69	
	Range	26–78	25–83	32–61	
ER status					0.014*
	+	64 (51.6%)	93 (60%)	12 (92.3%)	
	−	60 (48.4%)	62 (40%)	1 (7.7%)	
PR status					0.184
	+	21 (16.9%)	41 (26.5%)	3 (23.1%)	
	−	58 (46.8%)	67 (43.2%)	3 (23.1%)	
	N/A	45 (36.3%)	47 (30.3%)	7 (53.8%)	
HER2 status					0.648
	+	52 (41.9%)	57 (36.8%)	6 (46.2%)	
	−	71 (57.3%)	95 (61.3%)	7 (53.8%)	
	N/A	1 (0.8%)	3 (1.9%)	0 (0%)	
FNA axillary node	+	81 (65.3)	103 (66.5%)	8 (61.5%)	0.929
	−	43 (34.7%)	52 (33.5%)	5 (38.5%)	
Molecular subtype	DCIS	1 (0.8%)	4 (2.6%)	0	0.783
	IDC	115 (92.8%)	141 (91.1%)	11 (84.6%)	
	ILC	7 (5.6%)	8 (5.16%)	2 (15.4%)	
	Mucinous	1 (0.8%)	1 (0.6%)	0	
	Metaplastic	0	1 (0.6%)	0	

### Mammographic density and residual cancer burden

There was no significant difference in RCB score across the three MD groups (*p* = 0.192). 87 (29.8%) patients achieved a RCB score 0 indicating no invasive disease present in the breast or axillary lymph nodes. There was no significant difference in RCB score between MD category c and d (*p* = 0.155), MD category b and c (*p* = 0.409) and MD category b and d (*p* = 0.076). Similarly, the distribution of RCB class amongst the patients did not differ significantly across the three MD categories (*p* = 0.366) (Table 2).

**Table 2:** Residual cancer burden according to MD

MD		*b* (*n* = 124)	*c* (*n* = 155)	*d* (*n* = 13)	*p* value
RCB score	Median	2	2	3.068	0.192
	Mean	1.74	1.8	2.48	
	Range	1–2	1–2	0–3.852	
RCB class	0	40 (32.3%)	45 (29%)	2 (15.4%)	0.336
	1	14 (11.3%)	20 (12.9%)	0	
	2	43 (34.7%)	47 (30.3%)	5 (38.5%)	
	3	27 (21.8%)	43 (27.7%)	6 (46.2%)	

RCB score	*p* value
MD category c vs. d	0.155
MD category b vs. c	0.409
MD category b vs. d	0.076

* *p* = < 0.05

### Pathological complete response (pCR)

64 (21.92%) patients accomplished pathological complete response (pCR) following NACT in the current study (no evidence of invasive or in situ disease in the breast and axilla). The distribution of patient characteristics according to pCR is presented in Table 3. There was no statistical difference in age between the patients who achieved pCR and patients who did not achieve pCR (*p* = 0.957). Similarly, there was no statistical difference in menopausal status (*p* = 0.507) or MD category (*p* = 0.227) between those who achieved pCR and patients who did not. HER2 status differed significantly amongst those who achieved pCR and those who did not (*p* = 0.046) as patients who did not achieve pCR were more likely to be HER2−. Similarly, the proportion of patients with ER+ tumours differed significantly amongst the patients who achieved pCR and those who did not (*p* < 0.001) as patients who achieved pCR were more likely to have ER- tumours. Similarly, patients with PgR− tumours were more likely to achieve pCR than those with PgR+ breast cancer (*p* < 0.001). There was no difference in axillary FNA positivity between patients who achieved pCR and patients who did not achieve pCR (Table 3).

**Table 3:** Patient characteristics according to pCR

pCR		Yes *n* = 64	No *n* = 228	*p* value
Age (years)	Median	48	47	0.957
	Mean	48.86	50.21	
	Range	25–74	26–83	
Menopausal status	Pre	39 (61%)	144 (63.2%)	0.507
	Post	25 (39%)	84 (36.8%)	0.286
MD	B	32 (50%)	92 (40.3%)	0.227
	C	31 (48%)	124 (54.3%)	
	D	1 (2%)	12 (5.3%)	
RCB score	Median	0	2.415	
	Mean	0	2.349	
	Range	0	0–5.296	
RCB class	0	64 (100%)	23 (10.1%)	
	1	0	34 (14.9%)	
	2	0	95 (41.7%)	
	3	0	76 (33.3%)	
ER status				0.000*
	+	19 (29.7%)	150 (65.8%)	
	−	45 (70.3%)	78 (34.2%)	
PR status				0.001*
	+	8 (12.5%)	57 (25%)	
	−	46 (71.9%)	82 (36%)	
	N/A	10 (15.6%)	89 (39%)	
HER2 status				0.046*
	+	32 (50%)	83 (36.4%)	
	−	31 (48.4%)	142 (62.3%)	
	N/A	1 (1.6%)	3 (1.3%)	
FNA axillary nodes				
	+	37 (57.8%)	155 (68%)	0.130
	−	27 (42.2%)	73 (32%)	

* *p* = < 0.05

### Mammographic density (MD), residual cancer burden (RCB) and pathological complete response (pCR) for pre- and post-menopausal patients

183 pre-menopausal patients were included in the current study as defined by age (< 52 years). Of these there were 60, 111 and 12 patients in the BI-RADS MD category *b*, *c* and *d,* respectively. 109 post-menopausal patients were included in the current study (defined as age > 52 years) and there were 64, 44 and 1 patients in the BI-RADS MD category *b*, *c* and *d,* respectively. There were no patients in the BI-RADS MD category *a*. There was a significantly higher proportion of patients with BI-RADS category b in the post-menopausal cohort (*p* < 0.001) and a higher percentage of patients with BI-RADS c and d in the pre-menopausal cohort (*p* < 0.001, *p* = 0.024, respectively). There was no statistical difference between RCB scores and RCB class between the pre-menopausal and post-menopausal cohorts. Finally, there was no difference in pCR, ER, PgR, HER2, or nodal status between pre-menopausal and post-menopausal cohorts. The distribution of patient characteristics according to menopausal status is presented in Table 4.

**Table 4:** Patient characteristics according to menopausal status

Menopausal status		Pre-menopausal (< 52 years) (*n* = 183)	Post- menopausal (≥ 52 years) (*n* = 109)	*p* value
Age (years)	Median	38	63	< 0.001*
	Mean	38	62.84	
	Range	25–51	51–83	
MD category	B	60 (33%)	64 (59%)	< 0.001*
	C	111 (61%)	44 (40%)	< 0.001*
	D	12 (6%)	1 (1%)	0.024*
RCB score	Median	1.73	1.56	0.849
	Mean	1.84	1.83	
RCB class	0	58	29	0.366
	1	18	16	
	2	56	39	
	3	51	25	
pCR				0.507
	Yes	39	25	
	No	144	84	
ER Status				0.329
	+	112	57	
	−	71	52	
PR status				0.278
	+	38	19	
	−	73	54	
	N/A	72	36	
HER2 status				0.114
	+	116	54	
	−	59	54	
	N/A	8	1	
FNA axillary node	+	114	78	0.824
	−	69	31	

* *p* = < 0.05

### Factors associated with pCR and residual cancer burden

For both pre- and post-menopausal patients, there was no significant difference in RCB score across the three MD groups (pre-menopausal *p* = 0.374; post-menopausal *p* = 0.359). The distribution of RCB class amongst the pre-menopausal and post-menopausal patients did not differ significantly across the three MD categories (pre-menopausal *p* = 0.700; post-menopausal *p* = 0.421). Of the 64 patients who achieved pCR, 32 (50%) women expressed HER2, 28 (43.8%) had triple-negative breast cancer and 4 (6.2%) had ER receptor-positive/HER2-negative breast cancers. With respect to predicting pCR, the logistic regression model integrating the independent variables of hormonal subtype (ER positive/HER2 negative, ER positive/HER2 positive, HER2 enriched, Triple Negative), MD, histological grade and FNA positivity demonstrated good model fit (Hosmer and Lemeshow Test *p* = 0.748, Omnibus Tests of Model Coefficients *p* = 0.000). However, only breast cancer subtype was found to be a significant individual predictor of pCR. A binary logistic regression revealed that patients with HER2-positive breast cancer and triple-negative breast cancer are more likely to achieve pCR with an OR of 3.630 (95% CI 1.360–9.691, *p* = 0.010) and 2.445 (95% CI 1.131–5.288, *p* = 0.023), respectively (Table 5).

**Table 5:** Logistic regression analysis of factors associated with pCR (*N* = 64)

	Univariate analysis 0R	95% CI	*p* value	Multivariate analysis OR	95% CI	*p* Value
Grade 3 vs Grade1/2	0.468	0.231–0.952	0.036*	0.951	0.430–2.103	0.901
Nodal positivity	1.39	0.739–2.636	0.304			
ER+/HER2-	0.152	0.066–0.347	< 0.001*	0.443	0.186–1.054	0.066
ER+/HER2+	1.135	0.583–2.210	0.708			
ER−/HER2+	2.481	1.240–4.967	0.010*	3.630	1.360–9.691	0.010*
TNBC	3.052	1.683–5.534	< 0.001*	2.445	1.131–5.288	0.023*
BI-RADS MD b vs c/d	1.478	0.847–2.58	0.169			
BI-RADS MD c vs b/d	1.269	0.728–2.211	0.400			
BI-RADS MD d vs b/c	0.286	0.036–2.240	0.233			

## Discussion

The results of this study demonstrate that breast density did not appear to be an independent predictive marker of pathological response in the neoadjuvant setting. We found that oestrogen receptor positivity was significantly associated with BI-RADS category d, but MD was not an independent predictor of response to NACT.

These findings are concordant with the recent “NeoDense” study conducted by Skarping et al. in Sweden. Their prospective analyses of 200 breast cancer patients observed that whilst MD decreased in many patients during NACT it was not a predictive marker of pathological response to NACT [13]. Previously, the same group performed a retrospective study of 302 patients and concluded that women with BI-RADS d MD were less likely to achieve pCR post-NACT after adjusting for age and pre-treatment tumour characteristics. The results of both study sets were recently collated [14] and demonstrated that pre-menopausal women with category MD d were less likely to achieve pCR after NACT [14]. Conversely, a recent study of 442 patients by Di Cosimo et al. concluded that those with denser breasts showed an increased likelihood of pCR with odds ratio (OR) of 1.70, 2.79 and 1.47 for *b*, *c* and *d* categories. However, none of the MD categories were associated with pCR in the univariable logistic regression analysis and the results only proved marginally significant for MD category C rate using a fully adjusted model (OR c versus a: 2.79, 1.04–7.48) [15]. This distribution of patients in MD category a and d were significantly higher than those represented in the present study. An association between breast density and pCR/RCB score was not demonstrated in the current study.

This study did not chronologically track changes in breast density; however, there is substantial evidence to suggest that age and hormonal events may result in changes in MD. Menopause in particular results in a significant reduction in breast density [16]. Several studies have also investigated the change in breast density during neo- and adjuvant chemotherapy. A significant reduction in breast density post-adjuvant chemotherapy was demonstrated by Knight et al. and Sandberg et al. [17, 18]. Interestingly, a greater than 10% reduction in MD was associated with a significantly reduced risk of contralateral breast cancer compared to women with little or no change in MD. MD reduction is more prominent in younger pre-menopausal women receiving adjuvant chemotherapy compared to post-menopausal women [19]. The underlying biological explanation for reduced MD in pre-menopausal women during chemotherapy may be explained by the induction of amenorrhoea causing lobular atrophy [20].

A pCR was achieved in 21.9% of patients in this study, defined as no invasive or in situ residual disease in the breast and nodes. This strict definition of pCR was adopted as it can best discriminate between favourable and unfavourable patient outcomes [11]. Traditionally, patients with residual in situ disease or foci of invasive disease were considered to have achieved a pCR; however, a pooled analysis of 6377 patients treated with NACT reported an increased risk of relapse and death compared with the group of patients with stage ypT0 N0 breast cancer [11]. The pCR definition restricted to ypT0N0 showed the lowest adjusted HR for DFS and OS compared with the other definitions and should be more accurately adopted. Von Minckwitz et al. pooled analysis of seven NACT randomised controlled trials achieved an overall pCR rate of 15% which is significantly lower than the 21% achieved in this study. This can be explained by their historical patient cohort (1998–2006) and that the findings from this study resulted in a more careful patient selection for NACT. Results from this study suggest that patients with HER2-enriched breast cancer are almost four times more likely to achieve pCR. pCR is a surrogate endpoint for event-free and overall survival. Specifically, pathological complete response is associated with a 52% reduction in the probability of an event and a 64% reduction in the probability of death [21].

To the best of our knowledge this is the first study to examine the predictive value of MD using the RCB score in the setting of NACT. This index is subdivided into four classes with an increasing amount of residual disease: RCB 0 (pCR), RCB-I, RCB-II and RCB-III. An RCB score 0/class O refers to a complete pathological response with no evidence of invasive disease in the breast or axilla, the presence of in situ disease is not considered. 29.8% of patients in this study had Residual Cancer Burden score 0 which includes ypTisypN0 pathological stages. The RCB categories have been shown to correlate with long-term survival outcomes across triple-negative breast cancer (TNBC) and HER2 breast cancer subtypes and several clinical study groups, such as I-SPY [1, 2], ACOSOG (Z11103), CALGB (40601, 40603), NSABP (B-40, B-41) and ABCSG [22]. The RCB assessment is clinically useful as it is highly reproducible, with reproducible long-term prognostic significance [22]. Mammographic density was not associated with RCB assessment in this cohort of 292 patients.

There are several limitations to this study. The most noteworthy is the retrospective design of the study which limited study data. Menopausal status was estimated using the national average due to lack of individual patient information. Similarly, information on BMI, oestrogen exposure, parity and smoking were not available. Breast density was reported using the BI-RADS categorisation, whilst this is a validated and reproducible assessment of MD, a computer software programme for measurement of volumetric MD may have provided an additional objective categorisation. It is also worth noting that the number of patients with MD category d was low in this study (*N* = 13) which may influence the transferability of the results. Despite these limitations, our study had several strengths. The large sample size was reflective of a cohort of patients receiving NACT with detailed information of tumour characteristics. The proportion of tumour subgroups and pCR rate suggest that our study cohort is representative of the general patient group and offers external validity. The strict definition of pCR (ypT0N0) and the RCB score were both analysed to determine whether MD predicts response to NACT, neither of which were statistically significant.

In conclusion, our data suggest that whilst MD assessed at the time of diagnosis is significantly associated with oestrogen-positive breast cancer, MD is not an independent predictive marker of response to breast cancer NACT.

## Data Availability

Enquiries about data availability should be directed to the authors.
